# Kiel Gender Dysphoria Questionnaire (KGDQ): development and validation of a questionnaire for change-sensitive assessment of gender dysphoria

**DOI:** 10.3389/fpsyg.2025.1540500

**Published:** 2025-06-10

**Authors:** Antonia Möck, Inken Tödt, Mathis Landsberg, Alexander Pohl

**Affiliations:** ^1^Clinical Psychology and Psychotherapy, Institute of Psychology, Christian-Albrechts-University of Kiel, Kiel, Germany; ^2^Institute for Sexual Medicine and Forensic Psychiatry and Psychotherapy, ZIP GmbH, Kiel, Germany

**Keywords:** transgender, gender dysphoria, gender incongruence, transsexualism, mental health

## Abstract

We developed and evaluated the Kiel Gender Dysphoria Questionnaire (KGDQ), a tool designed to assess distress due to gender incongruence in individuals aged 18 and above with sensitivity to change. The 31 items of the questionnaire were generated through semi-structured guideline interviews with trans^*^ patients (*n* = 7) and experts (*n* = 5), which were analyzed using qualitative content analysis. Additionally, modified items from other questionnaires and the item collection of a working group were considered. Subsequently, the questionnaire was completed online by gender-dysphoric participants (*N* = 219). A principal axis analysis revealed a conceptually plausible three-factor structure with the subscales Alienation, Gender Role Pressure, and Body Dysphoria. All subscales demonstrated at least good internal consistency with α ≥ 0.80. A correlational comparison of two testing periods indicated high test-retest reliability (*r* = 0.84). The convergent validity with the Utrecht Gender Dysphoria Scale—Gender Spectrum and the divergent validity with the PHQ-9 module of the Patient Health Questionnaire were only partially demonstrated, as the predetermined thresholds were slightly under (*r* < 0.50) or overstepped (*r* > 0.40). The assessment of known-groups validity showed expected mean differences. The results suggest that the KGDQ is a reliable and valid instrument for capturing various aspects of gender dysphoria over time.

## Introduction

In Western societies, individuals are typically assigned to one of two gender categories—male or female—based on biological characteristics, usually at birth or even prenatally. The majority of the population perceives the assigned gender as congruent with their gender identity (the inner, deeply felt sense of belonging to a particular gender). For these individuals, Sigusch (1995) introduced the term cissexual. In contrast, trans^*^ individuals, however, do not identify with the assigned gender. According to the International Classification of Mental Disorders (ICD-10; World Health Organization [WHO], 2020), these affected individuals were classified as transsexual, which implied an understanding of them as mentally ill (F64.0, Transsexualism). Moreover, the ICD-10 was still based on a strictly binary conception, where individuals were presumed to transition either from female to male (trans^*^ men) or from male to female (trans^*^ women).

In the ICD-11 (Wiepjes et al., 2018) the incongruence between assigned gender and gender identity is referred to as Gender Incongruence, which is no longer classified under mental disorders but rather in a separate category Conditions related to sexual health. Furthermore, the definition has been expanded to include non-binary identities: “[…] a marked and persistent incongruence between an individual's experienced gender and the assigned gender […]” (World Health Organization [WHO], 2020, HA60). Therefore, the term trans^*^ describes the entirety of gender-diverse individuals - both binary and non-binary.

The distress often accompanying gender incongruence is referred to as Gender Dysphoria (Beek et al., 2015; American Psychiatric Association, 2013). Compared to the general population, the relative suicide rate among individuals with Gender Dysphoria is increased (e.g., Aitken et al., 2016; García-Vega et al., 2018; Wiepjes et al., 2018). The causes of this increase in the suicide rate are due to a higher prevalence of mental health disorders (Preuss, 2021) due to minority stress (Hendricks and Testa, 2012), lack of supportive structures (Poštuvan et al., 2019), among other factors. Among adolescents as well, an increased suicide risk is observed in trans^*^ compared to cis-identifying individuals (Aitken et al., 2016; Turban et al., 2020).

Fundamentally, gender dysphoria is best understood as a dynamic construct that can vary in intensity and severity over time and across social contexts (Galupo and Pulice-Farrow, 2020). To track changes across multiple specific parameters and to be able to assess the effects of interventions—whether psychotherapeutic or somatic—reliable and valid assessment tools are essential in clinical practice as well as in the field of research. In the context of the German Federal Social Court's ongoing jurisprudence, assessing clinically significant distress is essential for determining health insurance coverage obligations for the costs of (somatic) therapeutic interventions (Hamm and Sauer, 2014). Thus, assessing distress is equivalent with measuring gender dysphoria. However, there is a lack of change-sensitive and valid instruments enabling the assessment of gender dysphoria (Olson et al., 2011), which may be one reason for the heterogeneous results of previous therapy evaluation studies (Döhnert, 2020; Pöge et al., 2020). Widely acknowledged instruments, such as the Gender Congruence and Life Satisfaction Scale (Jones et al., 2018), may be used to assess overall life satisfaction while incorporating elements of gender congruence. At the same time, they also include a variety of non-specific aspects of well-being and psychological functioning—namely feelings of anxiety or low mood - that extend beyond the specific distress associated with gender dysphoria. Consequently, there is a need to develop such a specific instrument, as non-specific measures often capture only secondary symptoms and are therefore less suitable for tracking changes (Geissner and Schmitt, 2023). Henrich (2020, 2023) also demonstrated that there is no established consensus on sstandardized measurement procedures in the literature or international practice.

In English-speaking countries and the Netherlands, screening for gender dysphoria predominantly uses two established questionnaires: the Gender Identity/Gender Dysphoria Questionnaire for Adolescents and Adults (GIDYQ-AA, Deogracias et al., 2007) and the Utrecht Gender Dysphoria Scale (UGDS, Cohen-Kettenis and van Goozen, 1997). A modified version of the UGDS— the Utrecht Gender Dysphoria Scale—Gender Spectrum (UGDS-GS, McGuire et al., 2020)—has recently been developed, reducing the previously separate scales (for trans^*^ women and trans^*^ men) to a single version that encompasses the full gender spectrum. The GIDYQ-AA consists of subjective (e.g., satisfaction with assigned gender, item 1), social (e.g., similarities with men or women, item 11), somatic (e.g., body dysphoria, item 20), and legal (e.g., attempts to change legal gender in the past 12 months, item 23) domains. Items were allocated to these categories only by visual inspection, without empirical validation, and primarily capture trans-ident experiences. The name of the questionnaire alone suggests that it does not differentiate between transgender identity and Gender Dysphoria (i.e., the associated distress).

Both instruments are considered reliable and valid (Deogracias et al., 2007; McGuire et al., 2020). However, a study by Galupo and Pulice-Farrow (2020) showed that only about half of the trans^*^ participants felt their experiences of Gender Dysphoria were represented by the statements in the UGDS (54.0%) and GIDYQ-AA (52.5%), with particular criticism of the binary structure of these scales. UGDS and GIDYQ-AA are also only moderately correlated with each other (Schneider et al., 2016). This suggests that the two questionnaires capture different facets of transgender identity or gender dysphoria. Thus, using only one of these questionnaires may result in an invalid measurement of Gender Dysphoria.

Furthermore, neither the UGDS nor the GIDYQ-AA is likely sufficiently change-sensitive (e.g., Item 8 of the UGDS-GS [“Puberty felt like a betrayal.”]). Therefore, they may be less suitable for tracking progress over time, which is critical both in clinical practice and research (cf. Levine et al., 2023; van de Grift et al., 2017). Nevertheless, both instruments have been used in numerous studies, including for therapy evaluation (see, e.g. the review by Ludvigsson et al., 2023). In the context of evaluating progress during gender-affirming treatment, at a certain point, it becomes necessary to switch the questionnaire version (from male to female or vice versa). The timing of this switch is at the authors' discretion and varies widely. For instance, de Vries et al. (2014) argue that the version corresponding to the birth gender should be used even after hormone therapy, as hormone therapy, in their view, does not bring about sufficient physical changes. In contrast, van de Grift et al. (2017) switch to the social gender version after hormone therapy. Other authors do not specify the version used (e.g., Ristori et al., 2020). The UGDS-GS, as the newer version, has not yet been used for therapy evaluation. Additionally, it lacks standardization—particularly for the German-speaking context.

Overall, there is a gap in clinical and scientific practice regarding the change-sensitive measurement of Gender Dysphoria. Additionally, an international consensus of experts revealed a shift away from “established” questionnaires (Henrich, 2023), likely due to the lack of sufficiently change-sensitive tools.

## Objectives and research question

We present the development and validation of the Kiel Gender Dysphoria Questionnaire (KGDQ). This questionnaire aims to capture gender dysphoric distress in a change-sensitive manner. At the item level, we anticipate moderate item difficulty and discrimination values. With respect to the scale structure, we have no a priori expectations and therefore conduct an exploratory factor analysis. We further expect at least acceptable internal consistency, calculated using Cronbach's alpha. Our goal is for the KGDQ to demonstrate high test-retest reliability, sufficient convergent validity (measured by the correlation between KGDQ and UGDS-GS scores), and adequate divergent validity (measured by the correlation between KGDQ scores and PHQ-9 scores).

## Materials and methods

### Development of the KGDQ

In the initial phase, experts from the General Sexology and Sexual Therapy Working Group at the Institute for Sexual Medicine and Forensic Psychiatry and Psychotherapy (ISFP) at the Center for Integrative Psychiatry (ZIP) in Kiel (Germany) generated a pool of 86 potential items. This item pool was expanded to 255 items through three preliminary steps, which included a systematic literature review and semi-structured guideline interviews with seven patients and five experts. Redundant items were removed or merged, and the remaining items were revised to ensure change sensitivity and gender inclusivity, resulting in a set of 151 items. In discussion with clinical experts, this set was iteratively adjusted für clarity and content relevance reducing it to 33 items that were considered the most representative, comprehensible, and meaningful. In a following comprehensibility test with trans^*^ people (*n* = 8), two items were removed, leaving a final set of 31 items that were rated as straightforward and meaningful. The authors ensured that all items could be meaningfully answered by cisgender, transgender, binary, and non-binary individuals to enable known-groups analysis. To maximize user-friendliness, the questionnaire utilized a unipolar five-point Likert scale [from not at all (German: ü*berhaupt nicht*) to very much (german: *sehr stark*); Preston and Colman, 2000], where higher scores indicate greater distress.

### Validation of the KGDQ

The authors pre-registered the validation study on the AsPredicted platform prior to conducting it (pre-registration number: 126993; AsPredicted). The study itself was conducted online via LimeSurvey at two time points to assess test-retest reliability. The interval between the two measurement points was a minimum of 4 weeks. The initial survey took 15–20 min to complete. Participants were informed about the study background, goals, requirements, and procedures, as well as their rights and data protection measures. After providing consent, data was collected in a pseudonymized format. Participants generated a pseudonym to link the primary and retest data. In addition to sociodemographic data (assigned gender, gender identity, highest educational qualification, primary occupation, marital status), participants were asked about the presence of gender dysphoria and any gender-affirming measures (hormone therapy, surgeries, and legal gender change) they had undergone. They then completed the KGDQ, UGDS-GS, and PHQ-9 questionnaires. Participants could only proceed to the next page once all items on a questionnaire were completed, as there was no option to skip items. Lastly, participants had the opportunity to provide open feedback in a text field. During the second measurement period, only participants with gender dysphoria were surveyed and completed only the KGDQ; the UGDS-GS and PHQ-9 were not re-administered. Additionally, participants were asked whether they had come out in important areas of life or undergone gender-affirming interventions or a legal gender change between the two measurement points.

### Instruments

In addition to the KGDQ, self-assessment questionnaires were administered to assess current gender dysphoric symptoms and depressive symptoms: a German translation of the Utrecht Gender Dysphoria Scale—Gender Spectrum (UGDS-GS) and the Patient Health Questionnaire 9 (PHQ, Kroenke et al., 2001). As there is currently no German version of the UGDS-GS available, forward and backward translations were conducted based on Wahl et al. (2011). These two instruments were chosen due to their description in the literature as sensitive and economic measures with high validity (Gräfe et al., 2004; Löwe et al., 2004; Kroenke et al., 2001; Chen et al., 2023).

### Sample

Inclusion criteria for participation in the gender dysphoric sample were adulthood, sufficient German language proficiency, and current experience of gender dysphoria (“Do you currently experience gender dysphoria?”). Recruitment took place through various social media platforms, self-help organizations, qualified counseling centers, and contacts within LGBTQIA+ communities. The study was also promoted in treatment centers such as the ZIP in Kiel, Charité in Berlin, and “TransSuchtHilfe” by Kornelia Cost in Hamburg. Notably, the use of the/r/germantrans subreddit led to a rapid and substantial increase in the number of participants. The non-gender dysphoric sample was primarily recruited through social media (Instagram, groups in mobile messaging apps) and word-of-mouth.

**Figure 1:**
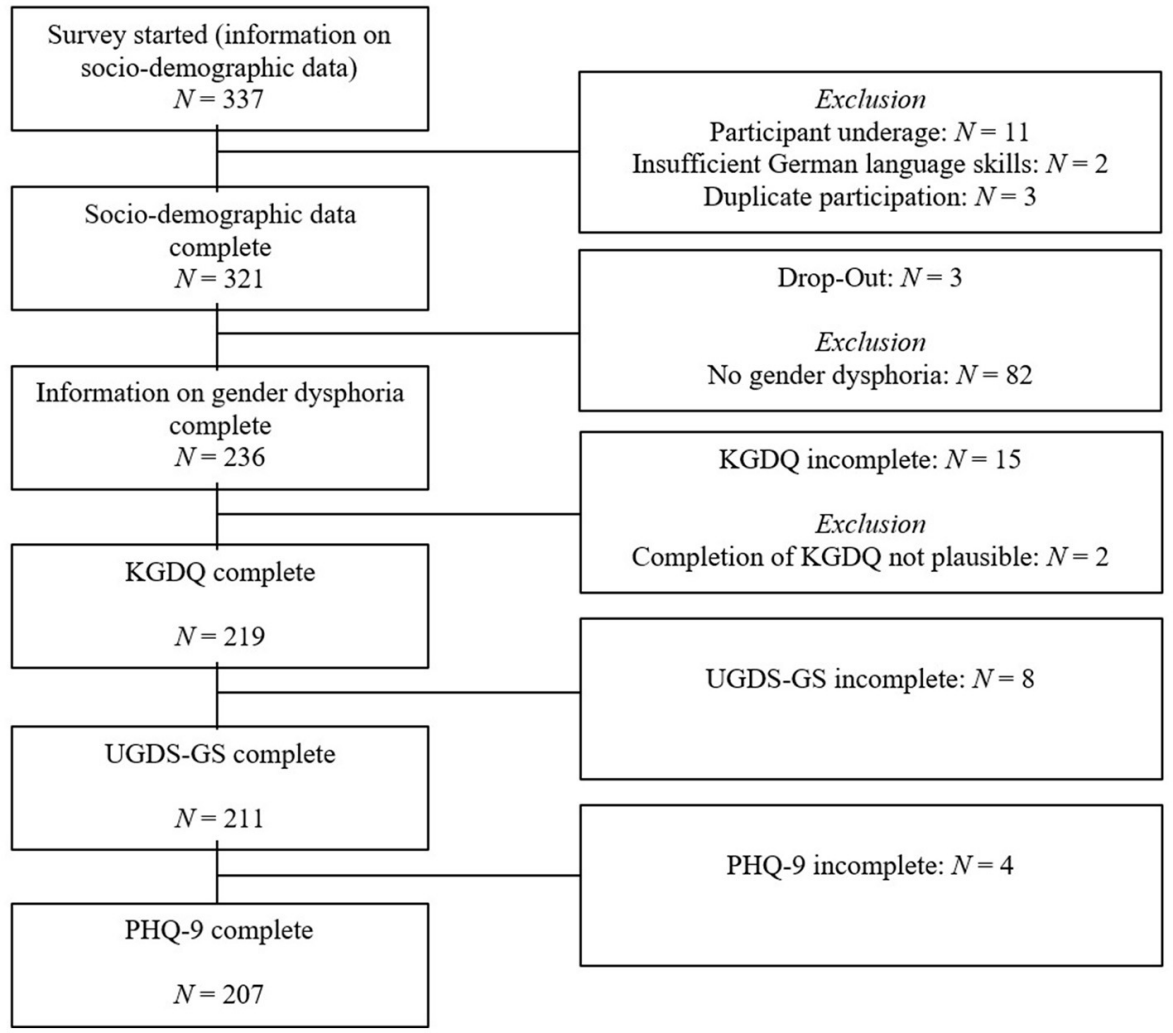
Dropout-analysis.

In total, demographic data were collected from 337 individuals (Figure 1). After excluding two individuals with insufficient German language proficiency, eleven minors, and three duplicate cases, 321 individuals with complete demographic data remained. Of these, 82 individuals who reported no current gender dysphoria were used as a control sample. The gender dysphoria status of three additional participants was incomplete, resulting in a total sample of 236 participants. The KGDQ was fully completed by 221 individuals; two cases were excluded due to apparently implausible responses (variance in answers of 0 or nearly 0), yielding a final KGDQ dataset of 219 cases. The UGDS-GS was fully completed by 211 participants, and the PHQ was fully completed by 207 participants. Of the 82 individuals without gender dysphoria, 79 completed the KGDQ in full, with two cases excluded due to implausible responses, resulting in a sample of 77 participants without gender dysphoria. For retest data collection, participants whose participation was at least 4 weeks prior were re-contacted by email. A total of 40 individuals provided complete demographic data for the retest. One minor and one individual with no history of gender dysphoria were excluded, and seven cases could not be matched, resulting in a retest sample of 31 participants.

The main analysis included data from the fully completed questionnaires of 207 participants aged 18 to 56 years (*M* = 26.28, SD = 6.94). More than half (57.99%) of respondents reported having experienced a mental disorder in their lifetime, most commonly depressive disorders (45.66%), anxiety disorders (26.03%), ADHD (20.55%), or autism (15.98%). Approximately half (47.49%) reported a current psychiatric diagnosis. Nearly three-quarters of participants had previously received psychotherapeutic support (counseling and psychotherapy; “Are you currently receiving psychotherapeutic support for your gender dysphoria?”) for gender dysphoria, and nearly half were receiving such support at the time of data collection. More than half (51.14%) were undergoing gender-affirming hormone therapy, with the majority (29.22%) having started it at least 12 months prior. Additionally, 13.70% had undergone gender-affirming surgery, and around a quarter (26.48%) reported a legal gender change. Since the inquiry about hormone therapies (HRTs) in this study was only directed at participants who answered positively to the question about psychotherapeutic support, the sample analyzed along the status of hormone therapy (*n* = 160) was limited to this subset of participants.

### Data analysis

All data analyses were conducted using *R* Studio (RStudio 2022.02.2; RStudio Team, 2022). For all administered questionnaires, the mean (M), standard deviation (SD), skewness, kurtosis, item discrimination, and item difficulty were calculated. Additionally, to assess the data suitability for exploratory factor analysis (EFA), the Bartlett's test, Kaiser-Meyer-Olkin coefficient (KMO), and Measure of Sample Adequacy (MSA) were calculated. To ensure high primary loading on the intended factor and low secondary loadings on other factors (Brandt, 2020), an oblique rotation based on the Oblimin method was applied, assuming that the various aspects of gender dysphoria are interrelated (Cooper et al., 2020).

Reliability was estimated using Cronbach's alpha and test-retest reliability, with confidence intervals constructed at the 95% level (see Gäde et al., 2020). To calculate test-retest reliability, a Pearson product-moment correlation between the total scores from the first and second test periods was calculated. According to Nunnally (1978), a reliability coefficient of *r* ≥ 0.70 is considered acceptable and thus set as a minimum threshold. According to Cicchetti (1994), for questionnaires potentially subject to fluctuations in the trait being measured, values as low as 0.40 can be considered acceptable, while values above 0.75 are regarded as excellent. Construct validity of the KGDQ was assessed by calculating divergent validity (Pearson product-moment correlation between KGDQ and PHQ-9) and convergent validity (Spearman rank correlation between UGDS-GS and KGDQ), alongside factorial validity. For convergent validity, the correlation should be *r* >0.50 according to Bühner (2011). For divergent validity, a coefficient of *r* < 0.40 is expected. Validity was further examined using a known-groups approach. A known-groups comparison of the KGDQ total scores between the gender dysphoric and non-gender dysphoric samples was conducted using the non-parametric Mann–Whitney U-test. For known-groups comparisons within the gender dysphoric sample, total score comparisons based on hormone therapy status and legal gender change were conducted using *t*-tests. The significance level for all tests was set at α = 0.05 (without α adjustment).

## Results

After evaluating the prerequisites, the dataset was deemed suitable for conducting a factor analysis. Both Horn's parallel analysis and the Scree criterion indicated a three-factor solution. All item difficulty levels fell within the targeted moderate range (*P*_*i*_ = 0.20–0.80), and all item-total correlations were above the minimum threshold (*r*_*i*_ ≥ 0.30). Based on the results of a principal axis analysis with oblimin rotation, five items were removed from the KGDQ, leaving the questionnaire with 26 items (Table 1). The corresponding metrics and detailed documentation of the item exclusion process can be found in the Appendix.

**Table 1:** Overview of the items included in the KGDQ.

**Items**
**Subscale 1: Alienation (*****n** =* **10)**
KGDQ28: feeling inferior because of your assigned gender
KGDQ31: experiencing too little interpersonal closeness because of your assigned gender
KGDQ18: feeling ashamed of the gender you were assigned at birth
KGDQ19: feeling lonely because of your assigned gender
KGDQ27: feeling wrong because of your gender identity
KGDQ23: feeling that no one fully understands you because of your assigned gender
KGDQ9: worrying that you might be rejected by others because of your assigned gender
KGDQ24: not being able to enjoy sexuality because of your assigned gender
KGDQ26: having little in common with people of your assigned gender
KGDQ13: having to give up something because of your assigned gender
**Subscale 2: Gender Role Pressure (*****n** =* **7)**
KGDQ21: being addressed with incorrect pronouns or titles
KGDQ11: having to categorize yourself according to your assigned gender (e.g., when filling out forms)
KGDQ14: being treated by others according to your assigned gender
KGDQ29: finding the first name given to you at birth inappropriate
KGDQ15: feeling pressured to behave in a “typical” way for your assigned gender
KGDQ22: feeling harassed by questions about your assigned gender
KGDQ17: having to justify yourself because of your gender identity
**Subscale 3: Body Dysphoria (*****n** =* **9)**
KGDQ5: perceiving “typical” physical aspects of your assigned gender in yourself
KGDQ6: perceiving a “typical” body shape for your assigned gender in yourself
KGDQ2: perceiving “typical” bodily functions for your assigned gender (e.g., erection/menstruation) in yourself
KGDQ20: wanting to hide your body, which is “typical” for your assigned gender, from others
KGDQ4: perceiving a “typical” facial structure for your assigned gender in yourself
KGDQ1: being dissatisfied with your assigned gender
KGDQ3: perceiving a “typical” appearance of your genitalia for your assigned gender in yourself
KGDQ8: perceiving a “typical” voice for your assigned gender in yourself
KGDQ7: perceiving a “typical” body size for your assigned gender in yourself

Items are translated into English for reasons of practicality and sorted by the magnitude of factor loading as in Table 2, each item is preceded by the sentence stem How much have you suffered from in the past 4 weeks?

A second principal axis analysis with oblimin rotation was then conducted. Following parallel analysis and the Scree plot, three factors were again extracted, which together explained 45.83% of the variance (Table 2). Eight items showed moderate item-total correlations (*r*_*i*_ ≥ 0.30), while the remaining 18 had high item-total correlations (*r*_*i*_ ≥ 0.50). All item difficulty levels remained within the moderate range.

**Table 2:** Eigenvalues and respective contributions of each factor to variance explanation (second exploratory factor analysis).

**Factor**	**Eigenvalues after rotation**	**Proportion of variance (%)**	**Cumulative variance (%)**
Factor 1	5.15	0.20	0.20
Factor 2	3.45	0.12	0.33
Factor 3	3.32	0.13	0.46

*N*= 219.

**Factor 1** was described as the subscale *Alienation*. This factor included ten items addressing the emotional experience of feeling detached from oneself and/or from others. **Factor 2** was labeled as the subscale *Gender Role Pressure*, with seven items capturing the stress arising from societal expectations regarding gender conformity. Lastly, **Factor 3**, identified as the subscale *Body Dysphoria*, included nine items reflecting distress and rejection associated with various physical attributes. The results of the second principal axis analysis of the KGDQ are shown in Table 3.

**Table 3:** Results of the second principal axis analysis of the KGDQ with oblimin rotation.

**Items**	**Factor loading**			***h* ^2^**	**Items**	**Factor loading**			***h* ^2^**
	**1**	**2**	**3**			**1**	**2**	**3**	
**Subscale1**					KGDQ29	0.13	**0.50**	0.17	0.43
KGDQ28	**0.78**	0.01	−0.03	0.59	KGDQ15	**0.37**	**0.49**	−0.16	0.41
KGDQ31	**0.76**	−0.11	0.06	0.57	KGDQ22	0.29	**0.48**	0.03	0.44
KGDQ18	**0.72**	−0.05	0.04	0.52	KGDQ17	0.14	**0.45**	0.04	0.29
KGDQ19	**0.70**	0.08	0.09	0.56	**Subscale 3**				
KGDQ27	**0.66**	0.12	0.01	0.53	KGDQ5	−0.08	0.10	**0.80**	0.65
KGDQ23	**0.54**	0.29	0.04	0.52	KGDQ6	0.02	0.05	**0.73**	0.58
KGDQ9	**0.53**	0.07	−0.04	0.29	KGDQ2	−0.05	0.08	**0.50**	0.26
KGDQ24	**0.52**	−0.22	0.27	0.41	KGDQ20	0.20	0.16	**0.49**	0.50
KGDQ26	**0.47**	0.25	−0.08	0.32	KGDQ4	0.23	0.07	**0.48**	0.44
KGDQ13	**0.45**	0.15	0.08	0.34	KGDQ1	0.22	0.14	**−0.47**	0.47
**Subscale2**					KGDQ3	0.27	−0.33	**0.45**	0.33
KGDQ21	−0.08	**0.74**	0.14	0.60	KGDQ8	0.06	0.29	**0.40**	0.38
KGDQ11	−0.05	**0.74**	0.09	0.58	KGDQ7	0.31	−0.14	0.28	0.22
KGDQ14	0.16	**0.66**	0.12	0.64					

*N*= 219, h^2^ = communality, factor loadings ≥0.35 are in bold. Items are sorted by the magnitude of factor loading. Subscale 1: Alienation (*n*= 10). Subscale 2: Gender Role Pressure (*n*= 7). Subscale 3: Body Dysphoria (*n*= 9).

Table 4 presents the descriptive statistics for the KGDQ (with 26 items). The average total score in the gender dysphoric group was *M* = 75.24 (SD = 21.51) with a range from 33 to 125. The total scores were approximately normally distributed, with a kurtosis of −0.89 and a skewness of 0.01. For the *Alienation* subscale with ten items, Cronbach's alpha was calculated as α = 0.89 [0.86; 0.91]. The Cronbach's alpha for the *Gender Role Pressure* subscale with seven items was α = 0.85 [0.82; 0.88], and for the *Body Dysphoria* scale (nine items) it was α = 0.83 [0.80;0.87]. For the gender dysphoric sample examined in the second testing period, an average total score of *M* = 67.74 (SD = 21.29) was recorded.

**Table 4:** Descriptive statistics of the gender dysphoric sample in the KGDQ.

**Variable**	**Sum scores**			**Response behavior**
	***M* (SD)**	**Empir. range**	**Theor. range**	***M*(*SD*)^a^**
Entfremdung	26.23 (14.21)	10–49	10–50	2.62 (1.42)
Geschlechterrollendruck	20.02 (9.97)	7–35	7–35	2.86 (1.42)
Körperdysphorie	28.99 (12.01)	10–44	9–45	3.22 (1.33)

*N*= 219.

^a^Mean and standard deviation averaged across individual items.

The average total score on the UGDS-GS was *M* = 75.51 (SD = 10.87), indicating potential ceiling effects. For the PHQ-9, an average total score of *M* = 11.99 (SD = 6.08) was calculated (range: 0–27). Within the gender dysphoric group, 8.22% showed minimal, 20.09% mild, 32.88% moderate, 21.00% moderately severe, and 12.33% severe depressive symptoms.

As expected, the non-gender dysphoric group had a significantly lower mean score on the KGDQ of 34.31 (SD = 11.54) compared to the gender dysphoric group. After graphical review, the distribution of total scores in the non-gender dysphoric group was classified as non-normally distributed, with a pronounced skew. A non-parametric distribution comparison using the Mann-Whitney U-test yielded a significant result (*W* = 684, *p* < 0.001, *d* = 2.11).

The Pearson product-moment correlation between the KGDQ total scores (among gender dysphoric participants) across the two time points was *r*_(29)_ = 0.84 [0.70; 0.92], *p* < 0.001. The Spearman rank correlation between mean total scores of the KGDQ and the UGDS-GS showed *r*_(209)_ = 0.47, *p* < 0.001. The Pearson correlation between the KGDQ and PHQ-9 was *r*_(205)_ = 0.46, *p* < 0.001.

### Exploratory analyses

When the Gender Role Pressure scale was excluded from the KGDQ, the correlation with the PHQ increased to *r* = 0.53 and with the UGDS-GS to *r* = 0.46.

### Known-groups validity

Participants who had undergone hormone replacement therapy (HRT) (*M* = 73.75, SD = 21.75) scored lower than participants who had not started HRT (*M* = 83.21, SD = 18.39), *t*_(51)_ = 2.63, *p* < 0.01, *d* = 0.45. An extreme group comparison showed that participants who had been on HRT for at least 12 months (*M* = 66.54, SD = 20.10) had significantly lower KGDQ-scores than those without HRT (*M* = 83.21, SD = 18.39), *t*_(105.35)_ = 4.54, *p* < 0.001, *d* = 0.86. Participants with a completed legal gender marker change (*M* = 66.26, SD = 20.07) also had significantly lower average KGDQ-scores than those without such a change (*M* = 78.48, SD = 21.14), *t*_(105.64)_ = 3.92*t, p* < 0.001, *d* = 0.59.

## Discussion

The experience of gender dysphoria is often accompanied by considerable psychological distress and is associated with an elevated likelihood of mental health disorders (Nieder et al., 2013; Romer and Möller-Kallista, 2022). The lack of sensitivity to change in existing measures of gender dysphoria is problematic, as it hinders the valid assessment of therapeutic progress.

### Study objectives and questionnaire development

The objective of this study was to develop and validate a German-language, change-sensitive questionnaire for assessing gender dysphoria. Validation efforts for the KGDQ included an exploration of its dimensional structure, which demonstrated that the questionnaire's development was successful. Item discrimination and difficulty analyses suggest the items are well-suited for measuring the severity of gender dysphoria. A conceptually coherent three-factor solution emerged, comprising the subscales **Alienation**, **Gender Role Pressure**, and **Body Dysphoria**.

- **Alienation** captures feelings of detachment from oneself or others. According to Cooper et al. (2020), who identified four sub-constructs of gender dysphoria in a systematic review, this subscale encompasses two aspects: first, the negative social consequences of expressing one's gender identity, particularly loneliness resulting from a perceived disconnection from others and communities (including frustrated needs for intimacy and sexuality); second, the internalized processing of these experiences, leading to a narrative of rejection followed by adverse emotional reactions as feelings of inadequacy or shame.
- **Gender Role Pressure** assesses the interplay between assigned gender, gender identity, and societal norms (Cooper et al., 2020). It covers situations where individuals feel compelled to conform to cisnormative, binary gender rules. Literature has identified gender role pressure as etiologically relevant to the development of gender dysphoria (e.g., Perry et al., 2019).
- **Body Dysphoria** evaluates dissatisfaction with specific physical characteristics. Given the highly individual nature of body dysphoria, the questionnaire addresses distinct attributes rather than relying on a single global item (Pulice-Farrow et al., 2020).

### Psychometric properties

The hypotheses regarding the KGDQ's internal consistency and test-retest reliability were confirmed. High Cronbach's alpha coefficients were obtained for all subscales (Gäde et al., 2020). Test-retest reliability analyses indicated strong reliability (*r* = 0.84), despite the dynamic nature of gender dysphoria. Notably, 41.94% of participants reported significant life changes, such as disclosing their identity to others, between measurement points, suggesting that individual variability did not undermine reliability.

### Convergent validity

Convergent validity, examined through the correlation between the KGDQ and the UGDS-GS, was deemed marginally insufficient. This discrepancy may be attributable to the differing temporal reference frames of the two instruments: while the KGDQ focuses on challenges experienced over the past 4 weeks, the UGDS-GS reflects lifetime experiences. Consequently, participants may have responded differently to similar items across the two measures, reducing correlation strength. Notably, the **Gender Role Pressure** subscale correlated less strongly with the UGDS-GS total score (*r* = 0.80) compared to the other KGDQ subscales (*r* = 0.85 and *r* = 0.90). Among the UGDS-GS items, only three (Items 2, 6, and 14) align with the concept of gender role pressure, potentially explaining the weaker convergent validity.

### Exploratory analysis and implications

The Gender Role Pressure subscale exhibited the lowest correlation with the KGDQ total score among the three subscales. This suggests that it may represent a distinct aspect of gender dysphoria, potentially reflecting more of an etiological rather than a symptomatic construct (Perry et al., 2019). An exploratory analysis excluding the Gender Role Pressure subscale from the KGDQ resulted in a correlation with the UGDS-GS exceeding the predetermined threshold of *r* = 0.05, indicating improved external validity. Additionally, the correlation between the UGDS-GS and KGDQ was significantly higher than previously reported correlations between the UGDS-GS and the GIDYQ-AA (cf. Chen et al., 2023).

These findings suggest that removing the Gender Role Pressure subscale could enhance the KGDQ's external validity and its utility for assessing gender dysphoria in clinical and research contexts.

The evaluation of divergent validity, assessed through the correlation between the KGDQ and the PHQ-9, indicated a correlation that was qualitatively too high. This suggests that the constructs of gender dysphoria and psychological distress are not entirely distinct. This overlap may stem from the substantial correlation between mental distress and gender dysphoria. By definition, gender dysphoria is experienced as distress by affected individuals (American Psychiatric Association, 2013), which inherently entails psychological burden (De Freitas et al., 2019). This relationship, particularly with depressive symptoms, was supported by the high prevalence of affective disorders in trans^*^ populations (Hanna et al., 2019; Hyde et al., 2014), as reflected in this study, where two-thirds of participants reported at least mild depressive symptoms on the PHQ-9.

The **Alienation** subscale specifically captures feelings of detachment, aligning closely with diagnostic criteria for depression in the PHQ-9. This overlap is further supported by findings linking discrimination experiences, internalized transphobia, and feelings of shame and anxiety with a heightened risk of depressive disorders (e.g., Chodzen et al., 2019; Kim et al., 2011).

Nevertheless, it remains necessary to assess depressive symptoms and gender dysphoria separately. While depressive symptoms can often be alleviated through targeted therapy, there is limited empirical evidence for the effectiveness of psychotherapeutic interventions in reducing gender dysphoria (Cuijpers et al., 2014; Murad et al., 2010). Additionally, not all depressive symptoms in trans^*^ individuals are mitigated by gender-affirming measures (Dhejne et al., 2016; Murad et al., 2010). Therefore, the AWMF-S3 guidelines (DGfS, 2019) recommend treating psychological disorders alongside gender-affirming interventions. Consequently, it is crucial to measure depressive symptoms and gender dysphoria separately, using instruments sensitive to changes over time.

### Known-groups analysis

The known-groups analysis further supports the validity of the KGDQ as a robust instrument. Significant differences were observed between gender dysphoric and non-gender dysphoric groups, as well as between treatment groups (e.g., with vs. without hormone therapy, and with vs. without legal gender recognition). These findings align with existing literature demonstrating that hormone therapy and legal gender recognition are associated with higher quality of life and reduced psychopathology (Baker et al., 2021; Hembree et al., 2017; Klein and Washington, 2023; Scheim et al., 2020).

### Sensitivity to change

The primary objective of developing the KGDQ was to create an instrument with high sensitivity to change, enabling intrapersonal comparisons. This capability facilitates the evaluation of progress, which is particularly valuable in clinical practice and research. By enabling both intergroup and intra-individual comparisons, the KGDQ allows for the monitoring of therapeutic outcomes over time. In this study, significant differences were already detected in a cross-sectional design between participants before and after physical transition measures. Future longitudinal studies with the KGDQ could further assess such changes. Sensitivity to change is a standard feature in assessing psychological distress, as demonstrated by established measures for affective disorders (e.g., Beck Depression Inventory-II [BDI-II], Beck et al., 2006) and anxiety disorders (e.g., the Anxiety Cognitions Questionnaire, Ehlers et al., 2001).

### Limitations and future directions

Despite efforts to adopt a sensitive, empathetic, and trans-affirming approach, the development, planning, and analysis of the study were conducted from a cisgender perspective (Galupo, 2017). Feedback from participants, particularly regarding item phrasing, was integral to refining the instrument. Criticisms included the complexity of language used and requests for greater precision in item wording. Future adaptations aim to enhance inclusivity, making the questionnaire accessible to individuals with lower educational attainment or non-native German speakers.

The sample was relatively young and highly educated, limiting its representativeness. The recruitment method, primarily through online communities such as the subreddit/r/germantrans, may have introduced selection bias. Regarding test-retest reliability, the small sample size (*N* = 31) is a notable limitation, restricting the interpretability of the strong correlation observed between initial and follow-up responses. Further validation in clinical settings and across diverse populations is planned, ideally involving cross-national studies.

For group comparisons based on HRT status, the sample size was reduced to *n* = 160, as this question was only posed to participants who had previously indicated that they were receiving psychotherapeutic support. Unsupervised use of hormonal treatments without medical indication could not be ruled out among the remaining participants. In future studies HRT status should not be linked with psychotherapeutic support.

Non-binary participants occasionally reported feeling underrepresented due to the questionnaire's focus on physical aspects, which were not sufficiently nuanced. While this feedback highlights an area for improvement, the diversity and heterogeneity of trans^*^ identities and experiences may make it challenging to comprehensively capture all perspectives within a single instrument (Pulice-Farrow et al., 2020).

## Conclusion

The KGDQ demonstrated reliability in assessing gender dysphoria. While it narrowly missed the thresholds for convergent and divergent validity, it is considered sufficiently valid based on the results of the known-groups analysis. In practice, the KGDQ is recommended for tracking changes in gender dysphoria over time. However, due to the reductionist nature of questionnaires, it should complement, rather than replace, in-depth discussions about individual experiences of gender dysphoria in therapeutic settings (Coleman et al., 2022).

In research, the KGDQ is well-suited for quantifying changes resulting from transition-related measures or other interventions. These data could further support advocacy for improved access to effective treatments. Future validation studies should include a broader range of clinical and cultural contexts, along with additional analyses, such as factor structure confirmation and exploration of auxiliary psychometric properties.

## Data Availability

The raw data supporting the conclusions of this article will be made available by the authors, without undue reservation.
