# Supplementary material for: Kiel Gender Dysphoria Questionnaire (KGDQ): development and validation of a questionnaire for change-sensitive assessment of gender dysphoria
Source: Front Psychol. 2025 Jun 10;16:1540500. doi: 10.3389/fpsyg.2025.1540500 (PMC12186655; doi:10.3389/fpsyg.2025.1540500)
Supplement: Supplementary file 1 [file Supplementary_file_1.docx]

Supplementary Material

# Supplement A

Results of the first principal axis analysis of the KGDQ with Oblimin rotation.

|  | Factor loading | | |  |  | Factor loading | | |  |
| --- | --- | --- | --- | --- | --- | --- | --- | --- | --- |
| Items | 1 | 2 | 3 | h^2^ |  | 1 | 2 | 3 | h^2^ |
| KGDQ28 | **.77** | .02 | .00 | .58 | KGDQ21 | -.10 | **.72** | .21 | .58 |
| KGDQ31 | **.75** | -.12 | .07 | .55 | KGDQ14 | .14 | **.65** | .17 | .63 |
| KGDQ18 | **.69** | -.08 | .09 | .50 | KGDQ15 | **.38** | **.50** | -.18 | .46 |
| KGDQ19 | **.67** | .08 | .09 | .56 | KGDQ22 | .27 | **.47** | .07 | .44 |
| KGDQ27 | **.66** | .12 | .01 | .53 | KGDQ29 | .11 | **.47** | .24 | .41 |
| KGDQ24 | **.53** | -.22 | .27 | .40 | KGDQ17 | .16 | **.46** | .04 | .31 |
| KGDQ9 | **.52** | .06 | -.01 | .29 | KGDQ12 | **.45** | **.46** | -.26 | .46 |
| KGDQ23 | **.52** | .27 | .07 | .51 | KGDQ5 | -.02 | .16 | **.73** | .61 |
| KGDQ13 | **.51** | .16 | .05 | .38 | KGDQ6 | .08 | .11 | **.65** | .54 |
| KGDQ26 | **.49** | .24 | -.08 | .34 | KGDQ1 | .23 | .14 | **.50** | .49 |
| KGDQ25 | **.48** | .10 | -.07 | .24 | KGDQ2 | -.04 | .10 | **.50** | .26 |
| KGDQ16 | **.46** | .00 | .30 | .44 | KGDQ20 | .22 | .18 | **.48** | .49 |
| KGDQ10 | **.38** | **.36** | .02 | .40 | KGDQ4 | .26 | .09 | **.46** | .25 |
| KGDQ7 | .34 | -.15 | .27 | .23 | KGDQ3 | .32 | -.34 | **.43** | .36 |
| KGDQ30 | .32 | -.02 | .17 | .18 | KGDQ8 | .08 | .29 | **.41** | .38 |
| KGDQ11 | -.08 | **.73** | .14 | .57 |  |  |  |  |  |

*Notes*. *N* = 219, *h^2^* = communality, factor loadings ≥ .35 are in bold. Items are sorted by the magnitude of factor loading.

Item adaptation: After a content re-evaluation, Item 30 was deemed insufficiently distinct and removed. Item 10 was excluded due to an ambiguous loading pattern (*λ_1_* = .38, *λ_2_* = .36) and potential redundancy with Item 9. Item 12 was removed due to its ambiguous loading pattern (*λ_1_* = .45, *λ_2_* = .46) and the negative phrasing “atypical”. Item 16 was excluded following a content review, as it was considered unsuitable for tracking progression. Item 25 was removed after a content review, as it could only be answered by binary individuals. Item 7 did not exhibit significant factor loadings (*λ_1_* = .34, *λ_2_* = -.15, *λ_3_* = .27), but could be clearly assigned to factor 3 (body dysphoria) in terms of content. Item 15 showed significant cross-loadings (*λ_1_* = .38, *λ_2_* = .50); in this case, the item was assigned to the factor with the highest loading.
